# Japanese Regulatory Framework and Approach for Genome-edited Foods Based on Latest Scientific Findings

**DOI:** 10.14252/foodsafetyfscj.D-21-00016

**Published:** 2022-12-23

**Authors:** Kazunari Kondo, Chie Taguchi

**Affiliations:** National Institute of Health Sciences, 3-25-26 Tonomachi, Kawasaki-ku, Kawasaki City, Kanagawa 210-9501, Japan

**Keywords:** genome-editing technologies, genome-edited foods, regulatory framework, Ministry of Health, Labour and Welfare, pre-submission consultation

## Abstract

The food supply system is facing important challenges and its sustainability has to be considered. Genome-editing technology, which accelerates the development of new variety, could be used to achieve sustainable development goals, thereby protecting the environment and ensuring the stable production of food for an increasing global population. The most widely used genome-editing tool, CRISPR/Cas9, is easy to use, affordable, and versatile. Foods produced by genome-editing technologies have been developed worldwide to create novel traits. In the first half of the review, the latest scientific findings on genome-editing technologies are summarized, and the technical challenge in genome sequence analysis are clarified. CRISPR/Cas9 has versatile alternative techniques, such as base editor and prime editor. Genome sequencing technology has developed rapidly in recent years. However, it is still difficult to detect large deletions and structural variations. Long-read sequencing technology would solve this challenge. In the second part, regulatory framework and approach for genome-edited foods is introduced. The four government ministries, including the Ministry of Environment, the Ministry of Agriculture, Forestry and Fisheries, and the Ministry of Health, Labour and Welfare (MHLW), started to discuss how the regulation should be implemented in 2019. The SDN-1 technique is excluded from the current genetically modified organism (GMO) regulation. The Japanese regulatory framework includes pre-submission consultation and submission of notification form. In the last part of this review, transparency of regulatory framework and consumer confidence were described. Since maintaining consumer trust is vital, transparency of regulatory framework is a key to consumers. The information of notification process on approved genome-edited foods is made public immediately. This review will help regulators build regulatory frameworks, and lead to harmonization of the framework between the countries.

## 1. Introduction

Genome-editing technologies enable the modification of one or more nucleotides in the target region using an enzyme that recognizes a specific base sequence in the genome. Classic examples of enzymes used for editing genomes are restriction enzymes, which are often used in biochemical experiments, including analyses of PCR products by agarose gel electrophoresis and analyses of the genome of a microorganism by pulsed-field gel electrophoresis. For example, the restriction enzyme *Eco*RI specifically recognizes GAATTC and cleaves the sequence after the first G (i.e., G/AATTC). Conventional restriction enzymes cleave only one predetermined sequence (e.g., GAATTC for *Eco*RI and CTCGAG for *Xho*I), whereas sequence-specific enzymes used in genome-editing technologies target any genome sequence. Genome-editing technologies have become popular because they are applicable for modifying most genomic regions. The technologies are now used in agricultural and medical fields^1^^,^^2^^)^. The clustered regularly interspaced short palindromic repeat (CRISPR)/CRISPR-associated protein 9 (Cas9) is useful for replacing mutated nucleotides causing genetic diseases with the original nucleotides^3^^)^.

There are three well-known sequence-specific enzymes in genome-editing technology. Zinc finger nucleases were first developed in 1996 and revolutionized genetic research, but the difficulty and cost of their construction prevented their general use in research. Second, the utility of the highly specific transcription activator-like effector nuclease (TALEN) was reported^4^^)^, and it has been used to study a variety of organisms from animals to plants^5^^,^^6^^,^^7^^,^^8^^,^^9^^)^. The enzyme has also been used to elucidate specific gene functions by gene disruption and to create new plant varieties. Third, CRISPR/Cas9, developed in 2012, has been a game-changer. CRISPR/Cas9 is surprisingly easy to use and inexpensive, which has led to its rapid adoption in many research fields.

Before the advent of genome-editing technology, it was necessary to use vectors derived from the soil bacterium *Agrobacterium tumefaciens* to insert target genes into the genome of plants. For example, herbicide-tolerant crops were created by introducing an herbicide resistance gene using the *A. tumefaciens* (i.e., GM plant). The first commercialized papaya ring spot virus-resistant papaya was created, and commercialized in 1996. Mutation breeding has been performed since 1940s. After irradiating crops with γ-rays, the desired crop traits were selected through laborious selection steps and backcrossing. In addition, backcrossing has been used to restore the genetic background of the original crop. Mutation breeding using γ-rays or chemical mutagens is not regulated in any countries all over the world. On the other hand, EU has regulated GM plants in a technology-based manner and USA in a product-based manner. The regulatory approach for genome-edited products is discussed in many countries.

At the 2018 OECD Conference on Genome Editing (Applications in Agriculture – Implications for Health, Environment and Regulation) in Paris, researchers and government officials discussed and shared information on recent technologies, applications and regulatory approaches in the member countries^10^^)^. Since then, the Ministry of Environment and the Ministry of Health, Labour and Welfare (MHLW) initiated discussions on the handling of genome-edited organisms in Japan.

This review summarizes the latest information on genome-editing technologies, clarifies the technical issues, and describes how Japan regulates foods produced by genome-editing technologies (hereinafter referred to as “genome-edited products” or “genome-edited foods”).

## 2. Genome-editing Technologies

**Fig. 1.**
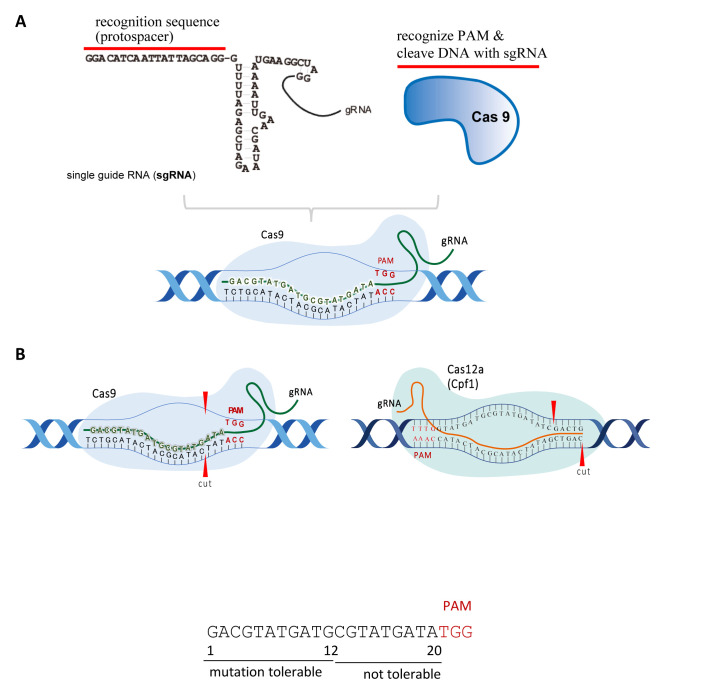
Overview of CRISPR/Cas9 and Cas12a (formerly known as Cpf1). ***A,*** gRNA and Cas9 protein complex induce DNA double-strand breaks at the target sequence. ***B,*** Cas9, and Cas12a have a 5′-NGG-3′ PAM and a 5′-TTTV-3′ PAM, respectively. Cas12a has a longer protospacer (21–24 bp) than Cas9 (20 bp). Cas9 and Cas12a create a blunt end proximal to PAM and a staggered end distal to PAM, respectively.

Genome-editing technologies have been used to add, remove, or substitute DNA sequences in specific genomic regions. CRISPR/Cas9 system is currently the most commonly used technique because it is easier to use and more accurate. An overview of CRISPR/Cas9 system is shown in **Fig. 1A**. The CRISPR/Cas9 system comprises guide RNA (gRNA) and Cas9 protein. The gRNA recognizes the 20-base sequence in one DNA strand and Cas9 recognizes an additional three bases after the 3′-end of the gRNA sequence in the other strand, which is called the protospacer adjacent motif (PAM) sequence. CRISPR/Cas9 system can recognize and cleave the target DNA sequence in just a few steps. First, Cas9/gRNA binds to the target site and unwinds the DNA duplex to form an R-loop. The Cas9/gRNA complex then cuts the double-stranded DNA at the target site^11^^,^^12^^,^^13^^,^^14^^)^. The first 8-12 PAM proximal base pairs form a seed region where mutations are less likely to be tolerated, and the PAM distal region is more mutation tolerant. To improve the sequence specificity, Cas9 orthologs were developed. Cas12a (Cpf1) was revealed to have higher specific cleavage activity^15^^,^^16^^,^^17^^)^. The protospacer motif of Cas12a (21–24 bp) is longer than that of Cas9 (20 bp). Cas9 and Cas12a create a blunt end proximal to PAM and a staggered end distal to PAM, respectively (**Fig. 1B**). Cas12a (Cpf1) is also used as a base editor (BE)^18^^)^.

**Fig. 2.**
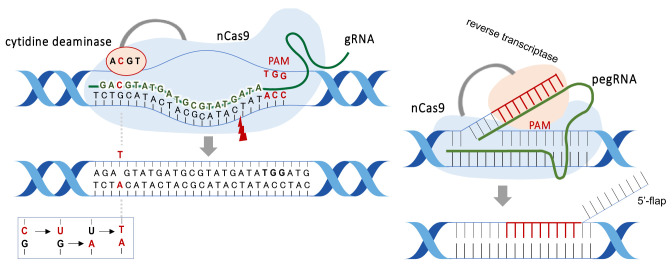
Overview of the cytosine base editor (CBE, *left*) and prime editor (PE, *right*). Both CBE and PE use nickase Cas9 (nCas9), which cleaves either strand of double-stranded DNA. CBE consists of gRNA and nCas9 fused to deaminase. Uracil glycosylase inhibitor (UGI) is also fused to nCas9, but it is not included to simplify the figure. The nCas9/gRNA complex recognizes the target sequence and induces a deamination reaction, resulting in the C-to-T change (for CBE). In contrast, PE comprises the prime editing guide RNA (pegRNA) and nCas9. nCas9 cuts one DNA strand, and reverse transcriptase polymerizes the DNA onto the nicked strand. The product of reverse transcription, an edited 3′ flap, can then be incorporated into the DNA duplex by competing with the original and redundant 5′ flap sequence. After the 5′ flap is excised and the edited strand is ligated, the non-edited complementary strand is replaced via DNA repair using the edited strand as a template.

Vectors encoding Cas9 protein and gRNA sequence have been used to introduce gRNA/Cas9. Recently, the Cas9 protein/gRNA complex can be introduced directly into cells^19^^)^. This method is expected to minimize adverse effects due to the shorter half-life of Cas9.-Genome-editing technologies can also enhance or suppress gene expression by inducing base substitutions or cytosine methylation without double-stranded DNA breaks. BE can substitute a single base by breaking one strand of DNA (Fig. 2, *left*). This technique was expected to suppress off-target mutations. The cytosine-based editor (CBE), which converts cytosine (C) to thymine (T), and the adenine-based editor (ABE), which converts adenine (A) to guanine (G), were developed^20^^,^^21^^,^^22^^,^^23^^,^^24^^,^^25^^)^. It was, however, later reported that unexpected genome-wide and transcriptome-wide mutations frequently occurred^26^^,^^27^^,^^28^^)^. The base editing technologies were found to induce unintended base substitutions in mouse and rice DNA^27^^,^^28^^)^. Therefore, ABE and CBE are still undergoing efforts to control unintended mutations. A new BE technique converting C:G to G:C was recently reported^29^^)^. Prime Editing (PE), which induces base substitutions using reverse transcriptase, was developed^30^^)^ (Fig. 2, *right*). Although PE is a more flexible technique than ABE and CBE, it requires a complex vector construct. PE was used to edit bases in rice and mice with up to 22% of editing efficiency, however, gRNA scaffold insertion and substitution were detected^20^^,^^31^^,^^32^^,^^33^^,^^34^^)^. PE3 (i.e., third-generation PE) is now reported to be more efficient^35^^)^.

CRISPR/Cas9 system has many tools, protocols, and experimental conditions. Therefore, a variety of results can be obtained with different genome-editing techniques. The frequency and outcome of genome editing at target and off-target sites vary widely depending on the experimental conditions.

## 3. Technical Issues Associated with Genome-editing Technologies

### 3.1 Categories of Genome Editing

**Fig. 3.**
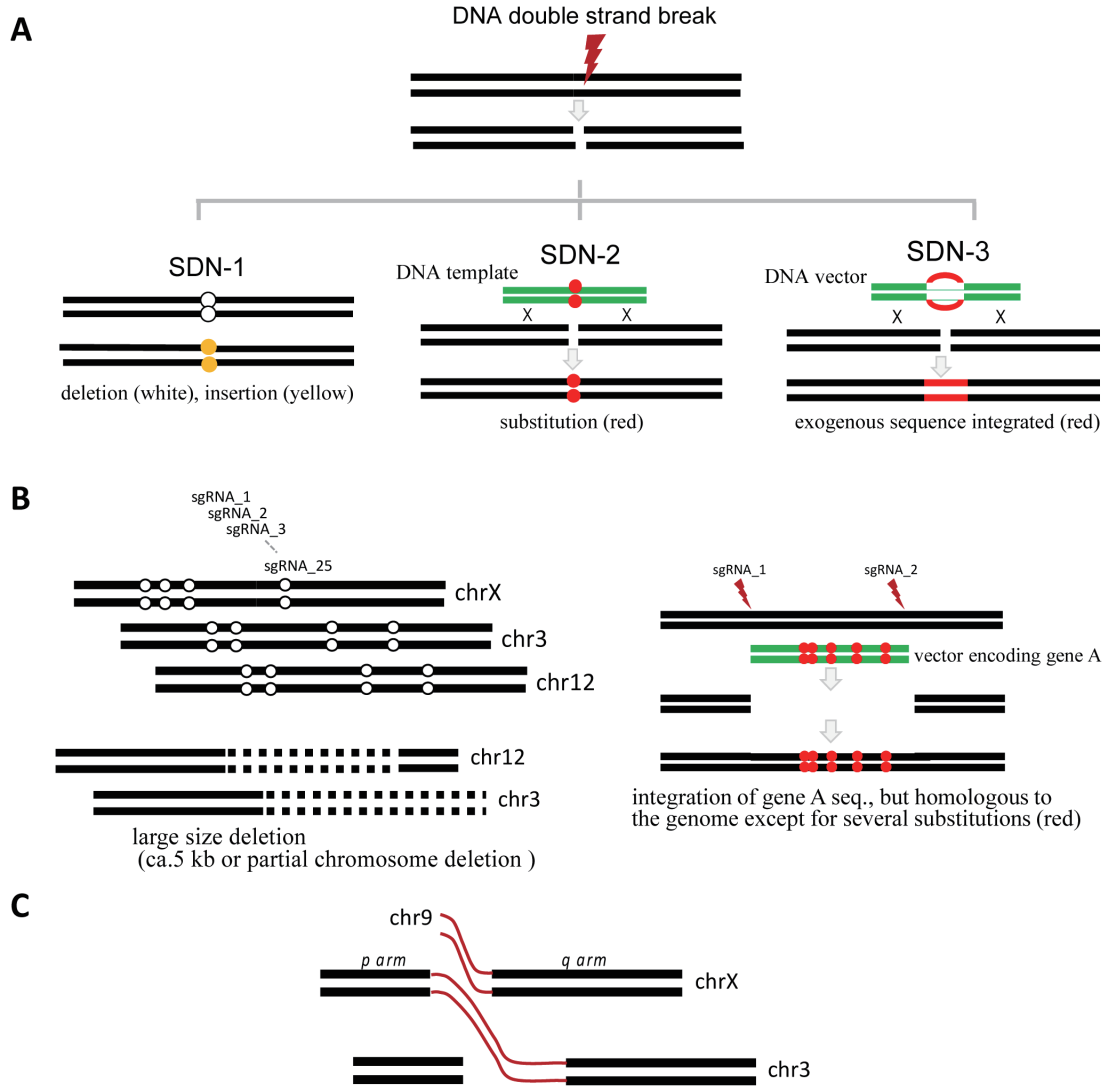
Types of genome-edited products and their alternatives. ***A***, Genome-edited products are classified into three categories (SDN-1, SDN-2, and SDN-3). SDN-2 and SDN-3 involve the use of a DNA template. ***B***, Simultaneous application of many gRNAs induces multisite deletions and large deletions, which can initiate translocations. In addition, large deletions can change chromatin structures (SDN-1, *left*). The integration of a homologous vector with five mutations creates multisite substitutions (SDN-1, *right*). This process is technically SDN-3, but the final product is more similar to the SDN-2 product. ***C***, Image of a translocation. Simultaneous multisite genome editing increases the chances of translocations.

Genome-editing technologies used to produce genome-edited foods are classified into the three categories of technique (site-directed nuclease (SDN)-1, SDN-2, and SDN-3) according to whether a DNA template is used or whether foreign DNA remains in the genome (**Fig. 3A**). Specifically, SDN-1, which induces the deletion of one to several nucleotides at the targeted sites, is associated with the non-homologous end-joining pathway. Single-base insertions also occur^36^^)^, whereas base substitutions are rare. Insertions and deletions (indels) may result in frame-shift mutations that alter the amino acid sequence. Therefore, the technical issues to be evaluated after the absence of foreign DNA in the genome has been confirmed include the effects of new open reading frames (ORFs) around the target site. The potential toxicity and allergenicity of candidate proteins encoded by the new ORFs must be evaluated. SDN-2 is used to introduce desired base substitutions. Because base substitutions do not lead to altered ORFs, the issues to be addressed, if any, may include whether the desired phenotype is due to the base substitution. If foreign DNA is integrated into the genome, the products are considered to be GM foods (i.e., SDN-3 products). Unlike SDN-1, SDN-2 uses a DNA template for introducing base substitutions. Although the donor vector can be integrated into the genome, the integration can be detected by next-generation sequencing (NGS) and conventional PCR analyses. However, the template DNA may induce base substitutions outside the target site (off-target effect) regardless of CRISPR/Cas9^26^^,^^27^^,^^28^^)^. The off-target base substitutions should be considered in SDN-2. The repair outcomes are sometimes the same between SDN-1 and SDN-2. Since SDN-3 products contain foreign genes or short sequences, it is necessary to investigate the impact in terms of what the inserted genes are.

### 3.2 Technical Issues

There are many available genome-editing technologies, however CRISPR/Cas9 system is usually used. For example, the simultaneous use of many gRNAs may result in large deletions and multiple indels across chromosomes (**Fig. 3B**, *left*), thereby increasing the chances of translocations (**Fig. 3C**). Although *in silico* searches are useful for detecting off-target sites, they cannot predict translocations. Conventional PCR amplifications followed by sequence analyses may reveal indels and substitutions, but not translocations. This limitation may be overcome by NGS, which can detect structural variations, including translocations. In addition, the integration of the homologous DNA donor vector with several base mismatches can generate sequences with several mutations in the genome using two gRNAs (gRNA-1 and gRNA-2; **Fig. 3B**, *right panel*). The resulting product technically belongs to the SDN-3 category, but it may be more appropriate to consider it as an SDN-2 product (i.e., not a GM product). This type of product will not be regulated as a genetically modified organisms (GMO) if the process (experimental condition) is not disclosed. An appropriate NGS analysis will reveal the random integration of the vector used into the genome, regardless of the technique (i.e., SDN-2 or SDN-3). Common technical issues for SDN-1, -2, and -3 include the formation of structural variations, including translocations (**Fig. 3C**). A conventional PCR targeting a 2-kb sequence is inappropriate for a target site with a 5-kb deletion. Without additional information, translocations cannot be detected by PCR analysis.

In 2012, European Food Safety Authority (EFSA) released the report, “Scientific opinion addressing the safety assessment of plants developed using Zinc Finger Nuclease 3 and other Site-Directed Nucleases with a similar function”, which indicated that the main difference between the SDN-3 technique and transgenesis is whether a predefined genomic region is targeted. The SDN-3 technique can minimize the damage associated with the disruption of genes and/or regulatory elements in the recipient genome^37^^)^. A report providing an overview of these genomic modifications was recently published^38^^)^. The GMO Panel suggested that compared with the effects of conventional breeding, genomic modifications induced by SDN-1, SDN-2, or ODM are not associated with additional risks. The GMO Panel also indicated that analyses of potential off-target sites are not useful for risk assessments because of the limited availability of fully sequenced plant genomes^39^^)^. The genome sequences of many organisms, including major crop species, are available in public databases, including OryzaGenome and GenBank (accession numbers start with GCA).

Draft genome sequences may be also useful for detecting mutations specific to genome-edited products because mutation analyses are generally performed to find mutations present only in genome-edited products, not in the original products. All mutations in the original products are subtracted from the corresponding genome-edited products using an appropriate pipeline, including GATK, BWA, and bcftools^40^^,^^41^^,^^42^^)^. Therefore, the mutations detected are specific to the genome-edited products. It is difficult to assess the impact of a mutation occurring at a particular position in a gene (functional annotation). There are issues associated with SDN-1 because more than 99% of the off-target mutations can be removed during the backcrossing over several generations^39^^,^^43^^)^. However, if backcrossing is performed for only two generations, 25% of the unintended mutations would theoretically remain in the genome. Is genome editing followed by two-generation backcrossing always enough to evaluate the safety? Genome-editing technologies are now widely used to modify diverse organisms (e.g., maize, wheat, rice, onion, apple, fish, algae, and mushrooms)^44^^,^^45^^,^^46^^)^. Some fruit trees are difficult to backcross.

There are generally no specific risks associated with the long-term presence of gRNA alone, whereas the presence of both gRNA and Cas9 may result in many unintended modifications. Recent research demonstrated that Cas9 alone can activate p53-inactivating mutations, leading to long-term irreversible changes in cultured cells^47^^)^. Moreover, CRISPR/Cas9 system induces complex rearrangements^48^^,^^49^^)^. Thus, elucidating the genome-editing outcomes and the subsequent breeding conditions is important.

### 3.3 Recent Technologies of Genome Sequencing and Introduction Methods

The recent rapid advances in sequencing technologies, especially the Nanopore sequencing technology that produces a single read comprising up to 4 Mb, enable the assembly of full genome sequences^50^^,^^51^^,^^52^^)^. The parallel use of Nanopore and Illumina sequencing technologies is becoming a powerful option for investigating genome sequences. In addition, DNA or RNA that encode Cas9 and gRNA, as well as the Cas9 protein/gRNA complex, is inserted into plant cells by methods using *Agrobacterium* species, biolistic bombardment, and nanoparticles. Plant viruses, polyethylene glycol, and *de novo* meristem induction may be applicable in the future^53^^)^.

## 4. Differences between Genome-editing Technologies and Mutation Breeding

Before GM plants were developed, mutation breeding via γ-irradiation and the use of chemical mutagens, such as ethyl methanesulfonate, was used to generate plants with novel traits. Specifically, γ-irradiation initially triggers DNA double-strand breaks at different sites depending on the radiation dose, which results in the induction of various mutations in different genomic regions (e.g., the deletion of a few bases or kilobases). Backcrossing and selection according to the phenotype are essential steps to establish the desired agricultural products. A recent study revealed that the final products have deletions shorter than 10 bp at a rate of nearly 2 × 10^−7^. The deletions of 1–16 bp were the most common mutations (63%) induced by γ-irradiation (80–300 Gy), followed by large deletions (9.4–130 kb; 17%) and single-base substitutions (13%). Medium-sized deletions (100 bp to 8 kb) were rarely induced by γ-irradiation^54^^)^. The rate of naturally occurring mutations was estimated to be approximately 7 × 10^−9^ base substitutions per site per generation^55^^,^^56^^)^, most of which are C:G to T:A transitions. The deletions exceeding 3 bp occurred at a rate of 0.5 × 10^−9^. The intergenic mutations (55%) were the most common, whereas non-synonymous mutations occurred less frequently (11%). Natural mutations and mutations induced by γ-irradiation mainly occur in introns and intergenic regions, slightly in exons.

Genome-editing technologies precisely induce DNA double-strand breaks at the target sequence at a much higher rate (approximately 0.2 or higher) than the spontaneous mutations described above (0.5 to 7 × 10^−9^). The frequency of double-strand breaks at off-target sites depends on the cell type. The use of ribonucleoproteins comprising gRNA and Cas9 can reduce the chances of off-target mutations compared to using plasmid DNA^57^^)^.

Mutation breeding by γ-irradiation can induce many random mutations. A γ-irradiation at a dose as low as 1 Gy can lead to 20–40 DNA double-strand breaks per cell^58^^)^, and thus a γ-irradiation at 300 Gy can initiate 6,000–12,000 DNA double-strand breaks per cell. Spontaneous DNA damage occurs in the order of 10,000–100,000 events per cell per day^59^^,^^60^^)^. These results suggest that many of the mutations induced by irradiation could be repaired inside cells.

## 5. Evaluation of Allergenicity and Toxicity

**Fig. 4.**
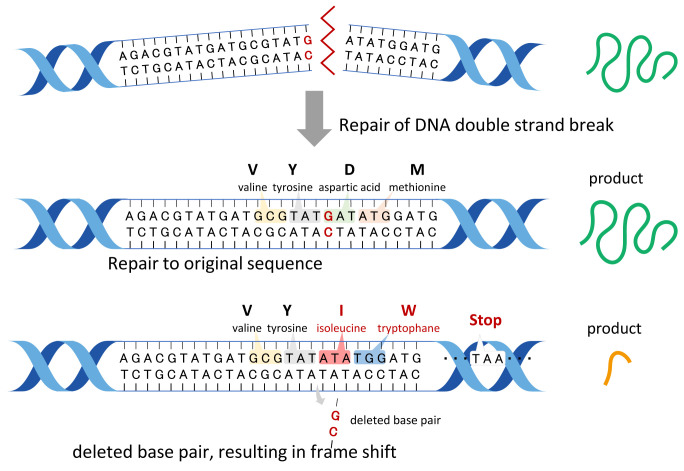
DNA repair mechanisms after a DNA double-strand break. Two typical pathways occur, repair (i.e., the original sequence is restored) and frameshift. A deletion introduces a premature stop codon as well as a frameshift leading to the formation of a different protein. Specifically, a non-functional truncated protein is produced. New ORFs derived from the frameshift must be analyzed in terms of whether they encode toxic and/or allergenic proteins.

To date, the safety of GM plants has been evaluated by examining the proteins encoded by the integrated genes and the potential proteins encoded by new ORFs in terms of their toxicity and allergenicity. Regarding SDN-1, foreign genes and sequences are not present in the final products (null segregant). However, new ORFs can emerge because of the introduction of indels in the coding region of an endogenous gene (**Fig. 4**). At present, the allergenicity and toxicity of the potential proteins are usually assessed using public databases, including COMPARE (https://comparedatabase.org), BLASTp, and UniProt. Briefly, the potential protein sequences are used as the query to search the databases for homologous proteins that are known allergens. Accordingly, novel allergens that are not homologous to already known allergens will not be identified by the database searches, and the current similarity research is, therefore, not a predictive method. Recently, a new method for assessing the allergenicity of proteins has been developed based on machine learning (ML) techniques^61^^,^^62^^)^. ML and deep learning techniques will be soon used to establish in silico allergenicity prediction methods.

What is the minimum amino acid length required to show allergenicity and toxicity? No standard has been established for assessing GMOs. Generally, proteins with fewer than 30 amino acids are ignored during the safety assessments. Here are some specific examples. Charybdotoxin (37 amino acids) is a neurotoxin present in scorpion venom. Pompilidotoxin in solitary wasp venom is only 13 amino acids long (RIKIGLFDQLSRL). Phalloidin and amanitin are cyclic peptides consisting of seven and eight amino acids, including D-amino acids. Among allergens, Api m 3 is considered to be the smallest allergenic protein (26 amino acids).

## 6. Recent Genome-sequencing Technologies

Many bioinformatics tools have been developed. For example, NGS is a powerful tool facilitating whole-genome analyses. To date, there are two technologies, short- and long-read sequencing technologies. The short paired-end sequencing technology in the Illumina platform is one of the most frequently used methods for analyzing genomes. The advantage of the long-reads in PacBio^63^^)^ and Oxford Nanopore^64^^)^ is that they can be read much longer at a time. Using PacBio and Oxford Nanopore sequencing, long repeated regions can be analyzed, such as telomeres, centromeres, and low-complexity regions. Software for mapping, variant detection, and annotation can be used to study the alterations in genomes at single-nucleotide resolutions. An in-depth NGS analysis can decipher structural variants, including complex DNA rearrangements and chromothripsis^65^^,^^66^^,^^67^^)^. However, the results vary among analysts. Standard guidelines for NGS analyses are urgently needed for risk assessment. Otherwise, the results of genome analysis may differ for each applicant.

## 7. Trends in Genome-edited Technologies and Products

**Fig. 5.**
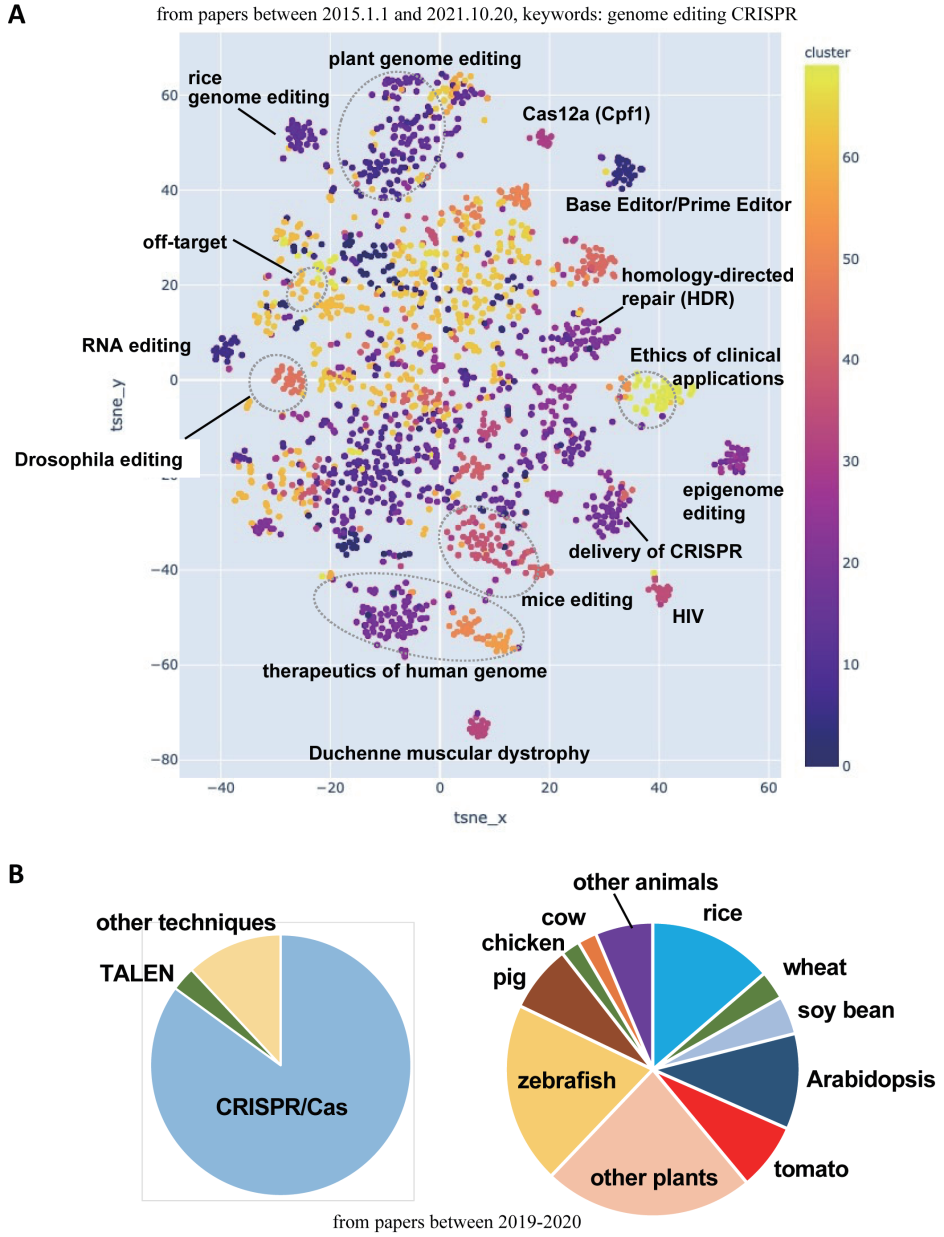
Cluster analysis of research articles on genome-editing technologies. ***A***, Therapeutics-related studies on Duchenne muscular dystrophy, HIV, and β-thalassemia are popular. Plant genome editing is also very popular, especially for rice. Technically, base editors, prime editors, and homology-directed repair (HDR) using DNA templates were thoroughly studied in the indicated period. Ethics regarding the use of genome-editing technologies for medical applications are currently being discussed. ***B***, CRISPR/Cas system is the most popular tool. Among plants, rice, tomato, and wheat/barley have been extensively studied. Among animals, pigs, chickens, and cows have been investigated.

In what fields have genome-editing technologies been studied? Recent trends were investigated through cluster analysis of published manuscripts describing research involving genome-editing technologies, especially CRISPR/Cas9. Research articles published between January 1, 2015 and October 20, 2021 were collected (7,840 articles in total) and then separated into clusters based on t-distributed stochastic neighbor embedding (t-SNE), which is a dimensionality reduction technique to visualize high-dimensional data in a two-dimensional figure (**Fig. 5A**). The articles describing applications in plants, especially rice genome editing, formed a large cluster. Studies on off-target sites, base and prime editing, epigenome editing, and editing using homologous recombination also formed a cluster, indicative of the substantial research that has been conducted.

From 2019 to 2020, CRISPR/Cas9 was the predominant tool (85%), followed by TALEN (3%). Rice (13%), tomato (7%), and wheat/barley (3%) were the most commonly studied plants, whereas pig (7%), chicken (2%), and cow (2%) were the main animal species that were investigated (Fig. 5B). The studies on plants included diverse fruits (e.g., strawberry, grape, apple, and orange). The studies on fish species were Atlantic salmon, Chinook salmon, channel catfish, loach, sea lamprey, and African cichlid. Zebrafish were well-studied fish species.

## 8. Ethics

Genome-editing technologies apply to the characterization of genomes of diverse organisms (e.g., fungus, plant, animal, and human). In clinical applications, *in vivo* and *ex vivo* somatic cell genome-editing techniques developed to correct mutated sequences responsible for human genetic diseases have been widely studied and may be useful if the benefits overweigh the risks.

Agriculturally important plants and animals are also being modified by genome-editing technologies. A variety of genome-edited plants would be commercially produced to increase yields and optimize the use of agricultural land (e.g., farmland affected by high salinity or drought). Genome-editing technologies could be used to achieve sustainable development goals, thereby protecting the environment and ensuring the stable production of food for an increasing global population. In recent years, there has been growing interest in genome-editing fish and other animals. However, animal welfare protocols and standards must be considered. In addition, several issues will need to be addressed, including whether the mass production of GM or genome-edited animals for food will be accepted by consumers. The increased use of genome-editing technologies in the agricultural field will depend on the understanding and acceptance of the general public. Genome-editing technologies may facilitate or enhance already controversial commercial practices in livestock breeding^68^^)^.

## 9. Regulatory Framework for Products Generated Using Genome-editing Technologies in Japan

The regulatory approaches for genome-edited products were studied in four different agencies, the Ministry of Environment, the Ministry of Economy, Trade and Industry (METI), the Ministry of Agriculture, Forestry and Fisheries (MAFF), and the MHLW. This chapter describes the results of the studies conducted by each ministry in chronological order.

### 9.1 Studies in the Ministry of Environment and the METI

On February 8, 2019, the Ministry of Environment released a notification regarding organisms obtained through genome-editing technologies based on the Cartagena Act (https://www.env.go.jp/press/2_2_%20genome%20editing_En.pdf; in Japanese). The Cartagena Act does not regulate genome-edited organisms generated using RNA and recombinant proteins (e.g., Cas9). Organisms generated by the transient expression of DNA construct encoding TALEN or CRISPR/Cas are also not covered by the Cartagena Act if they lack foreign genes or their fragments. Applicants are requested to provide the following information regarding genome-edited organisms:

1) Proof that the organism does not contain the remnants of extracellularly processed nucleic acids or their replicated products, as stipulated in the Cartagena Act

2) Taxonomical details regarding the modified organism

3) Method describing the use of genome-editing technologies

4) Modified gene and its functions

5) Changes in the target traits induced by the modification

6) Changes in non-target traits (i.e., other than those described in 5)

7) Purpose of use of the organism

8) Discussion regarding the possible effects on biological diversity when the organism is released

The METI requested that information be provided for use in open-field cultivation where containment measures have not been applied (https://www.meti.go.jp/policy/mono_info_service/mono/bio/cartagena/genome_yoryo.pdf).

### 9.2 Studies in the MAFF

On October 9, 2019, the MAFF released information regarding the regulatory procedures for organisms generated by genome-editing technologies. The procedures are applied to plants and animals in the agriculture and fisheries industries. Self-cloning organisms and naturally occurring organisms (i.e., phylogenetically the same or closely related species) were excluded. Applicants were required to consult with the appropriate agencies to determine whether the generated organisms were fallen into self-cloning or naturally occurring species based on scientific evidence.

### 9.3 Studies in the MHLW, and the Status of Genome-edited Foods Approved to Date

This review focuses on genome-edited foods, and therefore the results of scientific discussion in the MHLW are described in detail. The MHLW also released the Food Hygiene Handling Procedure (https://www.mhlw.go.jp/content/000550824.pdf) and started the pre-submission consultation and notification system on October 1, 2019. Applicants were requested to provide the following information for the pre-submission consultation:

1) Names of the item and breed as well as a summary of the current use and intended use of the food

2) Method describing the use of genome-editing technologies and details regarding the genomic modifications

3) Proof that there are no remaining foreign genes or their fragments

4) Proof that the confirmed changes in DNA do not result in the production of new allergens that adversely affect human health or increase the abundance of known toxic compounds

5) Whether the modification to increase or decrease specific components affects the metabolic system

6) Year and month of marketing (if decided)
Discussion of genome-edited foods on the Subcommittee

The MHLW Subcommittee on GM Foods, Newly Developed Food Committee of the Food Sanitation Council under the Pharmaceutical Affairs and Food Sanitation Council discussed the regulatory procedures for genome-edited foods as follows. First, the term “genome-editing technologies” was defined. As mentioned in “1. Introduction”, genome-editing technologies use sequence-specific enzymes to create new functions. The editing outcomes of genome-edited products are equivalent to those induced by conventional breeding and radiation breeding, which are exempted from the current GMO regulation. To date, the products have been classified into GM foods if foreign genes and their fragments exist in the final products based on the rule of recombinant DNA technology [included in the Specifications and Standards for Foods, Food Additives, etc. (the Ministry of Health and Welfare, Notification No. 370 of 1959)]. Therefore, the proof of the absence of foreign genes is a necessary condition for foods to be judged as genome-edited foods. Applicants should provide solid data confirming the absence of foreign genes and their fragments in the genome. Southern blot and PCR analyses have been used to produce the required proof, however, NGS is becoming a popular technique for that purpose. There was no discussion during the Committee members’ review process as to how short a gene fragment must be examined. In general, fragments shorter than 20-30 bases can be ignored unless they have any function.

Next, it was discussed what molecular characterization of genome-edited foods applicants are required to declare. In most cases, genome-edited foods are produced by introducing indels (SDN-1). As the resulting frameshift may generate a novel protein, toxicity or allergenicity of the protein must be appropriately examined. BLAST, UniProt, and COMPARE databases are commonly used. The same approach as the current GMO regulation was considered appropriate for evaluating the safety of genome-edited foods. The genetic stability of genome-edited organisms must be studied over a few generations at least.

Off-target effects were also discussed in the Committee. Genome-editing technologies work in a sequence-specific manner. Applicants can search for similar sequences to the target sequence in advance by using web-based *in silico* tools, such as CRISPRdirect (https://crispr.dbcls.jp) and Cas-OFFinder (http://www.rgenome.net/cas-offinder/). Applicants can design the specific target site. If *in silico* prediction tools detect off-target sites, PCR analysis can provide experimental evidence that the off-target mutation does not happen.

Finally, the regulatory approach of genome-edited products in which metabolic pathways have been altered was discussed. When one of the genes involved in a complex metabolic pathway that can give an impact on many surrounding genes was changed, applicants are required to submit a diagram showing which gene was altered on the metabolic map and its relationship to other related genes. Based on this information, the effect on the metabolic system needs to be considered. For example, fatty acid profiles would change and influence on human health when genes related to the formation of unsaturated fatty acid are genome edited. Therefore, the changes in gene products that exist in the metabolic pathway need to be investigated. After the scientific discussion, the regulatory procedures for genome-edited foods were released on September 19, 2019. The pre-submission consultation and notification system was started on October 1, 2019.

### 9.4 MHLW’s Regulatory Framework - Pre-submission Consultation and Notification in Japan

**Fig. 6.**
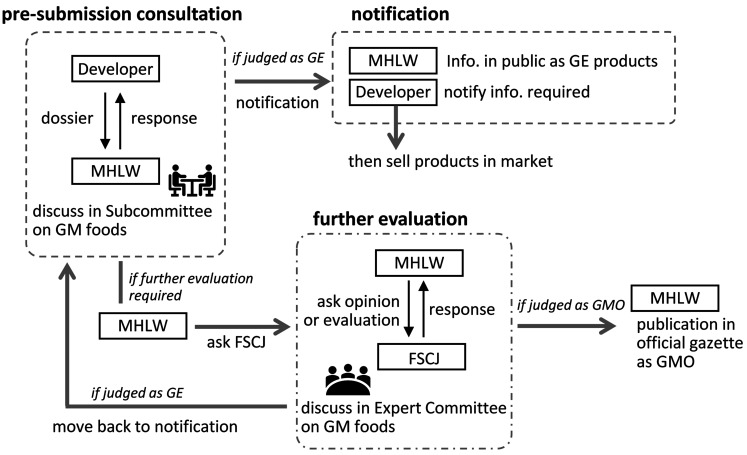
The MHLW’s regulatory framework - pre-submission consultation and notification system for genome-edited (GE) foods. Applicants first submit documents with the required information for GE foods. The MHLW asks the Subcommittee to confirm whether the submitted products comply with the established standards and regulatory procedures. If the products are considered to be GE products, then applicants can submit the required notification form to sell the products. Alternatively, products designated as GMOs must be carefully reviewed by the Food Safety Commission of Japan (FSCJ). *Subcommittee on GM foods in the MHLW has experts in toxicology, molecular cell biology, microorganisms, and plant and fish breeding. ** Expert committee on GMO in FSCJ includes experts from a wider range of fields. ***MHLW, Ministry of Health, Labour and Welfare. ****FSCJ, Food Safety Commission of Japan.

Applicants (developers) first submit a dossier containing the required information to the MHLW according to the regulatory procedures for foods produced by genome-editing technologies. The MHLW Subcommittee on GM Foods confirms the required information is included in the submitted documents. The most important criterion is the absence of any foreign genes or their fragments and backbone sequences of the vector used (null segregant) in the genome. If the Subcommittee members agree to judge the submitted product as a genome-edited product, the applicants can sell the product in the market after the proper notification form has been submitted. In addition, the MHLW will publish information explaining how the Subcommittee confirmed the product is safe. If the submitted product is considered to be a GMO, the MHLW will ask the Food Safety Commission of Japan (FSCJ) to assess its safety, similar to other GMOs. If the MHLW is unable to determine the safety of the submitted product at the Subcommittee level, even though the use of genome-editing technologies has been described and proof of the null segregant has been provided, the MHLW will consult with FSCJ to obtain a scientific evidence-based opinion. These steps in the regulatory framework are illustrated in **Fig. 6**.

Once a crop is designated as a genome-edited crop, it is considered the same as a crop obtained through conventional breeding. Therefore, backcrosses between genome-edited plants and conventional plants no longer need to be reviewed by the MHLW.

The regulatory approach in the framework described above is guided by the following principles.

#### • Based on scientific evidence

The Subcommittee members discuss the contents submitted by applicants from a scientific standpoint while also taking into consideration the effectiveness.

#### • Transparency and consumer confidence

The process that led to the decision to be considered notification for genome-edited foods is made public. If new findings on genome editing technologies are obtained, the framework will be reviewed as necessary. The MHLW will facilitate risk communication to promote public understanding and acceptance.

## 10. The Status of Genome-edited Foods Approved in Japan

To date, three genome-edited foods have been notified. Genome-edited tomato containing 4- to 5-fold more γ-aminobutyric acid (GABA) than wild-type tomato was first approved on December 11, 2020 and released in the market. The tomato glutamate decarboxylase gene *SIGAD3*, which negatively regulates the GABA level, was knocked out by the insertion of one base using vectors encoding Cas9 and gRNA. Southern blot and extensive PCR analyses covering the entire region of the vector used proved the absence of foreign sequences. The toxic glycoalkaloid tomatine was undetectable in the final products. The off-target analysis was performed using CRISPRdirect and Cas-OFFinder as well as PCR amplifications followed by Sanger-based sequencing. The mutations were detected at the target and the predicted off-target sites (55 sites) by the analyses. Whether new ORFs encode allergenic proteins was examined using COMPARE and FARRP (https://farrp.unl.edu) databases. Genetic stability was confirmed through three generations (https://www.mhlw.go.jp/content/11120000/000828873.pdf; in Japanese). Tomatoes with increased GABA content were approved in 2020. In 2020, two genome-edited fish were submitted to the MHLW. The MHLW Subcommittee has initiated discussions on the regulatory procedures for genome-edited fish. The current safety assessments of GM foods have been conducted only for plants. The discussion centered on how to consider an event. In GM plants, a plant of a single genomic pattern (i.e., event) can be evaluated. However, fish are bred in groups. After five-time discussions with the Committee members and external experts, the Subcommittee decided that a population with identical genetic changes at the target site on both alleles (e.g., deletion or insertion at the same position in diploid organisms) may be considered a single event for SDN-1 mutations. Two genome-edited fish species (sea bream and tiger pufferfish) were approved in 2021. Using Cas9 mRNA and gRNA, the myostatin gene *mstn* and the leptin receptor gene *lepr* were knocked out by 14- and 4-base deletions in sea bream and tiger pufferfish, respectively. Whole-genome sequencing and PCR analysis were conducted to reveal the absence of foreign sequences and off-target mutations. Protein allergenicity was investigated for the 10 and 61 newly emerged ORFs in sea bream and tiger pufferfish using FARRP and ADFS (https://allergen.nihs.go.jp/ADFS/) databases, showing no potential allergens. Red Seabream and Tiger Pufferfish with increased muscle mass were approved on September 17, 2021 and October 29, 2021. Thus, three genome-edited foods have been approved to date in Japan.

The number of plants and animals produced by genome-editing technologies is expected to increase. In assessing the safety of genome-edited foods, an appropriate analysis should be conducted to ensure that residual DNA sequences or changes in composition are not missed. In one case, the insertion of a plasmid DNA sequence was overlooked. In 2016, a hornless cow was developed using TALEN, and analysis immediately after its development confirmed the absence of foreign DNA^69^^)^. However, the Food and Drug Administration (FDA) subsequently found the presence of plasmid sequences in the genome-edited cattle^70^^)^. The developers reanalyzed the genomic sequence and confirmed the presence of the plasmid sequence^71^^)^. Finally, the developed cattle were incinerated. To accelerate the development of genome-edited plants, new techniques such as *de novo* meristem induction and the use of RNA viruses as vectors^72^^)^, will be applied, and large-scale genome-editing experiments will be conducted, which will allow for increasingly efficient genomic modifications.

## 11. Regulatory Framework in Other Major Countries

The USA updated the regulatory framework according to the “Memorandum on Modernizing the Regulatory System for Biotechnology Products” in 2015^73^^)^ and the “Executive Order (EO) on Modernizing the Regulatory Framework for Agricultural Biotechnology Products” in 2019^74^^)^. The Animal and Plant Health Inspection Service of the USDA issued the SECURE rule^75^^)^, which applies to genetically engineered organisms modified by genome-editing technologies and recombinant DNA technology. Notably, the SECURE rule does not apply to SDN-1 products, which means that SDN-1 is no longer genetically engineered plants. Genetically engineered animals are under the jurisdiction of the FDA. Whether the FDA continues to be involved in regulating GM plants though the consultation is unclear because the oversight of genetically engineered crops is now the responsibility of the USDA. However, the problems associated with genome-edited hornless cattle have reinforced the need for input from the FDA to ensure food safety.

In the EU, EFSA published a report on the assessment of the safety of plants developed through cisgenesis/intragenesis and zinc-finger nuclease-3 (corresponding to the current SDN-3) in 2012^37^^)^. In addition, the scientific opinion on SDN-1, SDN-2, and ODM was released. In the opinion, EFSA GMO panel did not identify new risks specifically linked to SDN-1 and SDN-2 techniques^38^^,^^39^^)^. In April 2021, the European Commission published the Commission Staff Working Document, “Study on the status of new genomic techniques under Union law and in light of the Court of Justice ruling in Case C-528/16”^76^^)^. This document states that products generated by genome-editing technologies can also be obtained through conventional breeding and cisgenesis. Thus, applying different levels of regulatory rules to products of similar risk levels may not be justified. Strict risk assessment guidelines are not suitable for case-by-case assessments and make it difficult to revise risk assessment requirements based on the basis of scientific progress. The final decision has not been made on regulatory procedures for genome-edited foods.

In the United Kingdom (UK), Food Standards Agency (FSA) plays in regulating food and feed and how FSA intend to develop a new regulatory framework to support the objectives of the Genetic Technologies (Precision Breeding, PB) Bill, which is currently progressing through the various stages of Parliamentary scrutiny. The regulatory framework would contain an authorization process for food and feed products produced using PB technology. This process includes safety, transparency, proportionality, traceability, and building consumer confidence.

Canada has a product-based risk assessment framework for plants with novel traits. This framework, developed by the Canadian Food Inspection Agency, applies to products obtained using genome-editing technologies^77^^)^, although the definition of “novel” is unclear. Health Canada announced that a new guideline will be issued soon.

In 2015, Argentina established a case-by-case consultation process to determine whether genome-edited products comply with the GMO regulations set by the Argentine Biosafety Commission (CONABIA). In Brazil, the National Biosafety Commission (CTNBio) approved Normative Resolution No. 16, which includes updated regulations for novel plant breeding technologies. Under these regulations, SDN-1 and SDN-2 products are evaluated as non-GMOs on a case-by-case basis^77^^,^^78^^,^^79^^)^.

## 12. Consumer Confidence

Maintaining consumer trust is vital. For example, FSA in the UK ensures consumer trust and interests by developing policies that put the consumer first (https://bills.parliament.uk/bills/3167). FSA commissioned research into consumer perceptions of genome-edited foods. The key findings suggested that there was very low knowledge of genome-edited foods^80^^)^. The situation is the same in other countries. Consumer acceptability of GMO has gradually increased. However, the ratio of acceptability is still low in Japan. The results of the web survey showed that consumer perceptions of “genome editing” was about 54% (unpublished data, MAFF). Twenty percent of the survey respondents were positive about promoting genome-edited high GABA tomatoes.

Transparency of regulatory framework is a key to consumer confidence. FSA reported that the regulatory framework must be clearly communicated and accessible to consumers and other stakeholders, and stakeholders must be involved in the development and operation of the framework to maximize open access to information^80^^)^. Most consumers feel that labeling should always inform the consumers of the presence of genome-edited ingredients using the full term “genome-edited”. A transparent regulatory framework, labeling system, and safety evaluation will provide consumers with confidence in genome-edited foods.

## 13. Conclusion

Foods produced by genome-editing technologies have been developed worldwide to create novel traits. Compared with the products of conventional mutation breeding, SDN-1 products do not have additional risks if genome-editing technologies are properly used, and the products are appropriately analyzed. SDN-2 products are similar to SDN-1 products, except for the use of a DNA template. The difference between SDN-1 and SDN-2 products cannot be distinguished by examining the final product alone. Genome-editing technologies currently include a variety of techniques, protocols, and experimental conditions. The outcomes also depend on cell types and organisms. The techniques used and the genome-edited outcomes should be considered carefully.

The consultation and notification system for genome-edited products in Japan requires information on molecular characteristics, proof of the absence of foreign DNA sequences, the results of off-target analysis, and the data for the quantitative analyses of toxicants and other chemical compounds. The submitted dossier is reviewed by the MHLW Subcommittee on a case-by-case basis. Once the Subcommittee judges genome-edited products, applicants can commercially produce and sell their products after submitting the required notification form. The content of the notification form is then published on the MHLW website.
